# A multimode metamaterial for a compact and robust dualband wireless power transfer system

**DOI:** 10.1038/s41598-021-01677-6

**Published:** 2021-11-11

**Authors:** Xin Jiang, Ramesh K. Pokharel, Adel Barakat, Kuniaki Yoshitomi

**Affiliations:** Graduate School of Information Science and Electrical Engineering, Kyushu University, Nishi-Ku, Fukuoka, 819-0395 Japan

**Keywords:** Electrical and electronic engineering, Energy harvesting

## Abstract

To release more flexibility for users to charge their portable devices, researchers have increasingly developed compact wireless power transfer (WPT) systems in recent years. Also, a dual-band WPT system is proposed to transfer power and signal simultaneously, enriching the system’s functionality. Moreover, a stacked metasurface has recently been proposed for a single band near-field WPT system. In this study, a novel multimode self-resonance-enhanced wideband metasurface is proposed for a robust dual-band WPT system, which significantly improves the performance of both bands. The size of the transmitter (Tx) and the receiver (Rx) are both 15 mm × 15 mm only. The proposed metasurface can improve efficiency from 0.04 up to 39% in the best case. The measured figure of merit (FoM) is 2.09 at 390 MHz and 2.16 at 770 MHz, respectively, in the balanced mode. Especially, the FoM can reach up to 4.34 in the lower mode. Compared to the previous state-of-the-art for similar applications, the WPT performance has significantly been improved.

## Introduction

Recently a compact wireless power transfer (WPT) system has become a hot topic because of the user's miniaturization and flexibility pursuit on portable devices. For example, in the medical domain, implanted medical devices (IMDs) such as pacemaker^1,2^, insulin pumps^3^, and so on^4^ are widely used, saving numerous people's life. However, when the IMD's battery inside a patient’s body runs out, as a solution for avoiding the battery replacement surgery, a wireless power transfer (WPT) system has been proposed in recent years^1,5^, which has a unique capacity of charging the devices from outside wirelessly. Moreover, compared with single-band WPT systems^6–9^, with the help of the dual-band WPT system, not only more power^10,11^ but also the information, i.e., for monitoring battery and patient's conditions by using each band, separately^12^ can be transferred on both bands.

To satisfy the need for these applications, the whole system must maintain adequate WPT efficiency at a long WPT distance by using the device compact enough to be implanted. With this purpose, recently, an artificial left-hand material with the dielectric characteristic of near-zero or negative permeability^13^ called metasurface has been widely used to improve the WPT efficiency^5,13–23^ enlarge the bandwidth of rectifier^5^, beamforming^24,25^ and empower the performance of the lateral misalignment^15,25^. Moreover, for a dual-band system, recent research^15^ employed a dual-band metasurface. Although a significant improvement was achieved at the lower band^15^, the efficiency improvement at the higher band seems much weaker than the lower band. Also, the employed metasurface was in a conventional 1D topology^5,13,15–22,25,26^, and more area seems to be exposed to the human body, which potentially causes health problems.

Hence, this research, inspired by the stacked metasurface^14^, a novel multimode self-resonance-enhanced wideband metasurface, has been proposed. Firstly, two types of self-resonance-enhanced wideband metasurfaces are proposed, which are estimated to be effective on each band, respectively. The metasurface that works on the lower band consists of split-ring resonators (SRRs) stacked. This type of metasurface is represented as a 180° metasurface because of the opposite position of the gaps on each ring. Similarly, the metasurface that works for the higher band comprises SRRs where each ring gap is kept in the same direction. This type of metasurface is represented as a 0° metasurface. The unit cells of both metasurfaces are stacked vertically to save not only the area but also more power that can be enforced at the desired direction^14^ compared with a conventional 1-D planar metasurface^5,13,15–22,25,26^. Then, these two types of metasurfaces are arranged together on each side. Also, three modes (higher, lower, and balanced mode) are generated because of the topology of the metasurface after the combination. In the balanced mode, the improvement can be estimated on both bands.

## Dual-band WPT system

**Figure 1:**
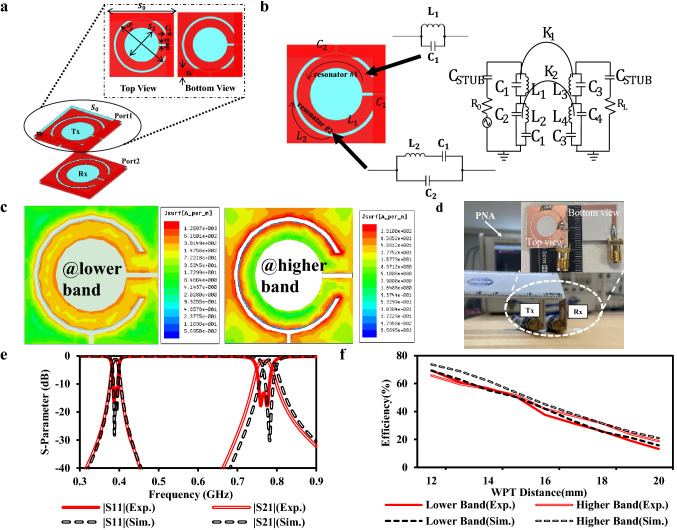
Configuration and experiment setup of the proposed DGS-based dual-band WPT system. (**a**) System layout. (**b**) Equivalent circuit. (**c**) Current distribution when resonance frequency goes to lower band (left) and higher band (right) to evaluate the isolation performance of the overlapping rings. (**d**) Experiment setup. (**e**) Comparison between simulation and experiment results of S-parameter at a WPT distance of 12 mm. “Exp.” stands for the experiment result. “Sim.” stands for simulation result. (**f**) Simulated and measured efficiencies when the WPT distance changes from 12 to 20 mm.

Figure 1a shows the configuration of the proposed dual-band WPT system. Compared to the conventional coil systems^8,9^, defected ground structure^27^ (DGS) is proposed to minimize the layout of the resonators. A conventional DGS exhibits band stop filter (BSF) characteristics at its dominant mode. DGS can be viewed as an inductor by loading with appropriate capacitance, as shown in Fig. 1b. The combination of DGS inductance and the loading capacitance releases more freedom on adjusting the geometry^27^. The admittance (*J*-) inverter method^28^ has been applied to optimize the value of the elements of the admittance matrix in Fig. 1b.

Moreover, two DGS resonators are overlapped, shown in Fig. 1b, to save the layout further. Also, as the EM simulation result shown in Fig. 1c, few mutual interferences on each band resulting in an excellent isolation performance. Figure 1e shows the simulation and the experiment result of the S-parameter at the WPT distance of 12 mm. Moreover, the WPT efficiency ($\eta$) can be depicted by^14^:

1 $$\eta ={|{S}_{21}|}^{2}\times 100\%$$

Based on Eq. (1), the simulation and experiment results on different WPT distances are illustrated in Fig. 1f. The WPT efficiencies drop dramatically from the distance of 16 mm, and few efficiencies can be observed when the WPT distance is above 20 mm. Therefore, the stacked metasurfaces are proposed in the next section for better performance at the longer distance and compact WPT system.

## Self-resonance-enhanced wideband stacked metasurface

**Figure 2:**
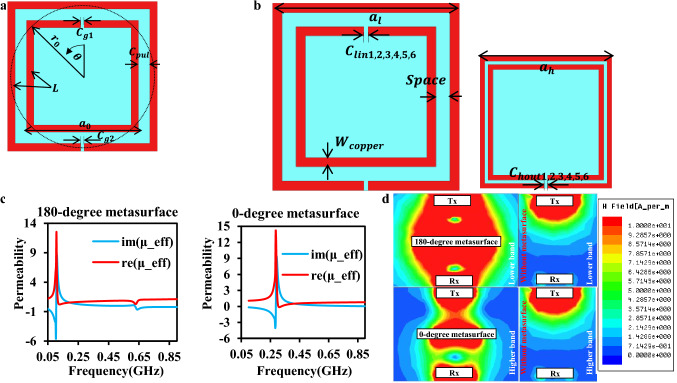
Wideband metasurface. (**a**) Structure of the rectangular SRR and its approximation to a circular ring shown in the dash line. (**b**) 0° SRR metasurface (left) and 180° SRR metasurface (right). (**c**) Permeability in boths cases and (**d**) Comparison between EM simulation of the system with/without metasurface at a WPT distance of 40 mm, which was chosen because the efficiency is almost zero at this point without a metasurface.

For the proposed dual-band WPT system, two types of self-resonance-enhanced wideband stacked metasurface are proposed in this study. Compared to the conventional metasurfaces^5,13,15–22,24–26^, which are all confined with the 1-D topology, the stacked metasurface offers more flexibility on the topology, making it possible for more arrangement for different types of metasurfaces altogether. Although the dual-band metasurface with a significant improvement on the lower band was proposed in the former work^15^, only a slight improvement was observed on the other band. Therefore, based on the stacked metasurface^14^ mentioned above, two types of metasurfaces that can be effective at the lower or higher band are first proposed using the rotational SRR. In the near-field WPT system, the proposed metasurface must satisfy two conditions for realizing the enhancement effect^14^. The first condition is that the real part of the permeability in the z-direction should be less than 1 ($(real\left({\mu }_{z}\right))<1$) so that the magnetic field can be enforced toward the Rx. At the same time, the imaginary part should be near zero for avoiding loss caused by the metasurface itself. Besides the permeability, the whole WPT system is supposed to be impedance matched^20^ in the same resonance frequency for each element (Tx, metasurface, and Rx). The self-resonance frequency on a SSR of each metasurface can be reasonably estimated by^29^

2 $${f}_{0}=\frac{1}{2\pi \sqrt{2{r}_{0}L\frac{{(\pi +k)}^{2}-{\theta }^{2}}{2(\pi +k)}{C}_{pul}}}$$

where, as shown in Fig. 2a, $L$ is the total inductance of the SRR, ${C}_{pul}$ is the per unit length capacitance, and $\theta$ is the rotation angle. Also, in Eq. (2), $k=\frac{{C}_{g}}{{r}_{0}{C}_{pul}}$, ${C}_{g}$ is the gap capacitances, and ${r}_{0}$ is the average radius of the inner rings^30^.

Hence, based on Eq. (2), 180° metasurface and 0° metasurface, as shown in Fig. 2b, are proposed for each band separately. The permeabilities of these two metasurfaces are estimated the same way as the former work^14^ in Fig. 2c to satisfy two conditions on each band. According to the electromagnetic (EM) simulation shown in Fig. 2d, the magnetic fields are enforced into the z-direction on each resonant frequency using 180° and 0° metasurface, respectively, resulting in the coupling improvement between the Tx and the Rx.

## Multimode self-resonance-enhanced wideband metasurface

**Figure 3:**
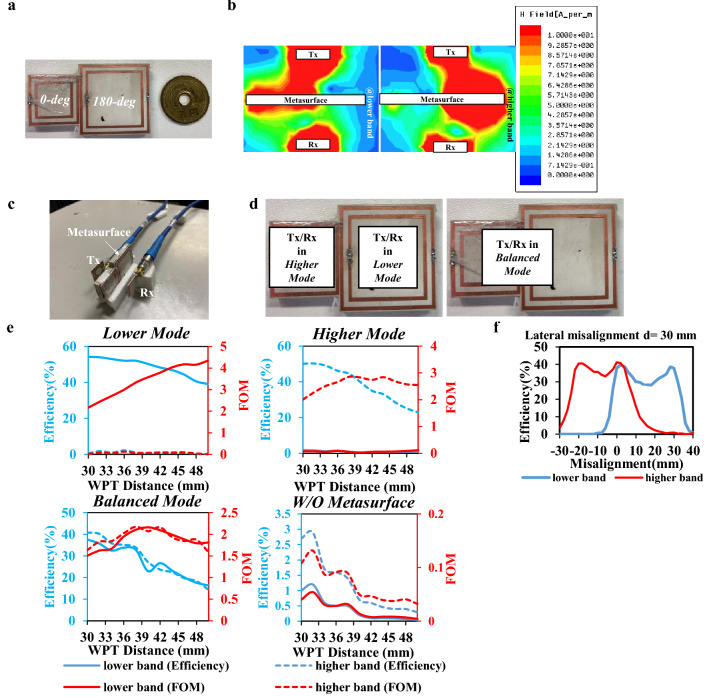
Proposed multimode self-resonance-enhanced metasurface. (**a**) Proposed metasurface. “0-deg” stands for the 0° metasurface and “180-deg” stands for 180° metasurface. (**b**) EM simulation at lower and higher band, respectively when WPT distance = 40 mm. (**c**) Measurement setup (**d**) Definition of three mode. The white boxes stand for the location of the Tx (Rx). When Tx/Rx put toward the 0° metasurface, the whole system will be in lower mode and verse sersa. (**e**) WPT efficiency (blue line) and FOM (red line) at lower band (solid line) and higher band (dash line) in different modes and without metasurface (W/O Metasurface). (**f**) Lateral misalignment from − 30 to 40 mm at the WPT distance of 30 mm.

The metasurface shown in Fig. 3a is constructed by combining the 180° and 0° metasurface on each side. However, when these two types of metasurface are arranged together, cross-polarization occurs, resulting from the strong interference between these two metasurfaces^31^. As a result, a massive reduction in efficiency occurs. As a solution, the polarization of the field through each SSR can be easily changed by modifying the position of each SSR^32^. Consequently, as the EM simulation result shown in Fig. 3b, the magnetic field at each band is enforced into the z-direction concurrently and separately, resulting in a significant improvement on both bands.

Figure 3c shows the experiment setup of the proposed dual-band WPT system using the proposed metasurface. Due to the topology of the proposed metasurface, three working modes are defined in Fig. 3d based on the intersection area. When the 180° metasurface covers more against the Tx and Rx, the efficiency increases at the higher band, called higher mode, and vice versa. On the other hand, when the proposed metasurface is at the middle of the proposed WPT system, the enhancement effect on both bands is balanced, of which mode is named as balanced mode.

Figure 3e shows the experiment and simulation result of the WPT efficiency. In the lower mode, the WPT efficiency reaches 39.1% at the lower band in the distance of 50 mm. In the higher mode, the WPT efficiency is 33.0% at the higher band in the distance of 44 mm. In the balanced mode, the WPT efficiencies are 26.7% at the lower band and 27.6% at the higher band at the WPT distance of 42 mm. Also, the lateral misalignment performance at the distance of 30 mm is shown in Fig. 3f. Because of the impedance mismatching problem^20^ that occurred when the Tx is moved horizontally, the efficiency on both bands firstly goes down, goes up, and goes down again. These measured results show the necessity of further improvement of impedance matching in metasurface, which will be studied soon using a machine learning technique. Moreover, considering the size, distance, and WPT efficiency, which are three perquisites for the wireless IMDs, the performance of this WPT system using metasurface can be evaluated by Eq. (3), which is named as figure of merit (FoM)^33^.

3 $$FOM=\frac{{\eta }_{peak}\times {d}^{2}}{{{A}_{Rx}}^\frac{2}{3}{{A}_{Tx}}^\frac{1}{3}}$$

where $d$ is the WPT distance and ${A}_{Tx},{A}_{Rx}$ are the area of Tx and Rx, respectively. Based on the FoM, compared with the previous state-of-the-art, Table 1 implies a significant improvement on both bands on the balanced mode. Also, in the lower mode, the WPT performance on the lower band is far better than the previous works^15^.

**Table 1:** Performance comparison with state-of-the-art designs.

WPT	Area of Tx/Rx (mm^2^)	Operation	Metasurface	Distance (mm) (maximum performance)	Normalized distance $\left( {\frac{d}{{\sqrt[4]{{A_{{Tx}} A_{{Rx}} }}}}} \right)$	η	FoM
^9^	290 × 290 × π	Single band	Without	875	1.7	69.40%	2.01
^15^	390 × 390/130 × 130	Dual band	Without	200	0.89	26.9%/16.8%	0.213/0.133
			With			42.38%/24.5%	0.336/0.194
^22^	150 × 150	Single band	Without	200	1.3	10.7%	0.190
			With			54.9%	0.976
This work	15 × 15	Dual band	Without	15	1	50.4%/51.5%	0.504/0.515
			Balanced mode	42	2.8	26.7%/27.6%	2.09/2.16
			Lower mode	50	3.33	39.1%/0.09%	4.34/0.01
			Higher mode	44	2.93	0.7%/33.0%	0.06/2.83

## Conclusion

To enhance the proposed dual-band WPT system, a novel multimode self-resonance-enhanced wideband metasurface has been proposed. As a necessity to be applied to the compact devices, i.e., IMDs, the compactness is realized by overlapping DGS resonators, avoiding interference between each band. For each band, because the resonance frequency of the SRR varies from each ring’s angle, the 180° and 0° metasurface are proposed, which are effective and have a self-resonance at each band of the WPT system, respectively. Then the multimode metasurface is proposed by arranging these two types of metasurfaces together. To fix the cross-polarization problem resulting from the combination of the metasurfaces, the directions of each metasurface are re-adjusted.

As a result, the proposed metasurface improves the WPT efficiency up to 39.2% (from 0.04% without metasurface) in the best case. The balanced mode metasurface improves the WPT efficiency on both bands simultaneously. Moreover, the proposed metasurfaces are also equally effective in improving the lateral misalignment in a similar trend. These performances result in the FoM of the proposed WPT system above two in all cases, which overwhelms state-of-the-art systems as illustrated in Table 1.

## Methods

### WPT system using DGS

Based on the equivalent circuit shown in Fig. 1b, the resonant frequencies of each band are ${f}_{1}=\sqrt{(4{\pi }^{2}{L}_{1}{C}_{1}{)}^{-1}}$ and ${f}_{2}=\sqrt{\frac{{C}_{1}+{C}_{2}}{4{\pi }^{2}{L}_{2}{C}_{1}{C}_{2}}}$. Here, ${C}_{1} \left(10 pF\right),{C}_{2} (2 pF)$ are the capacitances of each gap and ${L}_{1,}{L}_{2}$ are the inductances of the inner and outer ring, respectively. The Rogers 3003 substrate (dielectric constant = 3, substrate thickness = 0.762 mm, copper thickness = 17 µm) has been used for both simulations and fabrication.

### Self-resonance-enhanced wideband metasurface

**Figure 4:**
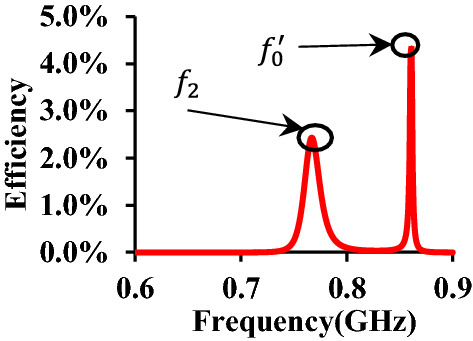
Efficiency at the higher band when ${f}_{2}\ne {f}_{0}^{^{\prime}}$. ${f}_{2}$ stands for the WPT resonant frequency at the higher band. ${{\varvec{f}}}_{0}^{\boldsymbol{^{\prime}}}$ stands for the metasurface self-resonance frequency. When ${f}_{2}\ne {f}_{0}^{\mathrm{^{\prime}}}$, the WPT efficiency decreases.

To the author's knowledge, most of the research regarding the WPT system using metasurface mainly focuses on the permeability of the metasurface itself. However, when it comes to dual-band WPT systems, even if the wideband metasurface, of which the characteristics of near-zero permeability can be estimated in the wide range of the frequency, the significant improvement can still not be realized on two bands at the same time. We found that the efficiency curve, shown in Fig. 4, exhibits two peaks when the proposed metasurface is inserted inside the whole system during the EM simulation process. The reason for the peak on the right side (${f}_{0}^{^{\prime}}$) occurs due to the resonance of the metasurface itself. In the proposed WPT system, each cell of the proposed metasurface can be seen as a passive element^20^. Therefore, when the passive elements (in this study is each cell of the metasurface) are added to the whole system, the impedance of the whole system ($\widehat{{\varvec{Z}}}$) can be written as^20^:

4 $$\widehat{{\varvec{Z}}}={\varvec{Z}}+j{\varvec{C}}+{{\varvec{R}}}_{L}$$

here, ***Z*** refers to the unloaded impedance matrix, which can be described as^20^:

5 $${\varvec{Z}}=\left[\begin{array}{ccc}\begin{array}{c}{{\varvec{Z}}}_{{\varvec{T}}{\varvec{x}}}\\ {{\varvec{M}}}_{{\varvec{T}}{\varvec{x}},{\varvec{p}}}^{\mathrm{T}}\\ {{\varvec{M}}}_{{\varvec{T}}{\varvec{x}},{\varvec{R}}{\varvec{x}}}^{\mathrm{T}}\end{array}& \begin{array}{c}{{\varvec{M}}}_{{\varvec{T}}{\varvec{x}},{\varvec{p}}}\\ {{\varvec{Z}}}_{{\varvec{p}}}\\ {{\varvec{M}}}_{{\varvec{p}},{\varvec{R}}{\varvec{x}}}^{\mathrm{T}}\end{array}& \begin{array}{c}{{\varvec{M}}}_{{\varvec{T}}{\varvec{x}},{\varvec{R}}{\varvec{x}}}\\ {{\varvec{M}}}_{{\varvec{p}},{\varvec{R}}{\varvec{x}}}\\ {{\varvec{Z}}}_{{\varvec{R}}{\varvec{x}}}\end{array}\end{array}\right]$$

here, ${{\varvec{Z}}}_{{\varvec{T}}{\varvec{x}}}$,${{\varvec{Z}}}_{{\varvec{p}}}$, and ${{\varvec{Z}}}_{{\varvec{R}}{\varvec{x}}}$ stands for the unload impedance of the Tx, the passive elements, and the Rx, respectively. ${{\varvec{M}}}_{{\varvec{T}}{\varvec{x}},{\varvec{p}}}$, ${{\varvec{M}}}_{{\varvec{T}}{\varvec{x}},{\varvec{R}}{\varvec{x}}}$, and ${{\varvec{M}}}_{{\varvec{p}},{\varvec{R}}{\varvec{x}}}$ stands for the mutual-impedance resulting from the coupling between two of these groups together.${{\varvec{M}}}_{{\varvec{T}}{\varvec{x}},{\varvec{p}}}^{\mathrm{T}}$ stands for the transpose of ${{\varvec{M}}}_{{\varvec{T}}{\varvec{x}},{\varvec{p}}}$. Also, the ***C ***contains the capacitors etched on the Tx, passive elements, and the Rx, which can be described as

6 $${\varvec{C}}=\left[\begin{array}{ccc}{{\varvec{C}}}_{{\varvec{T}}{\varvec{x}}}& & \\ & {{\varvec{C}}}_{{\varvec{p}}}& \\ & & {{\varvec{C}}}_{{\varvec{R}}{\varvec{x}}}\end{array}\right]$$

Therefore, the other peak of the efficiency emerges at the same place where the power loss (${P}_{l}$) is minimum. The power loss can be depicted as^20^:

7 $${P}_{l}= \frac{1}{2}{{\varvec{i}}}^{H}Re({\varvec{Z}}){\varvec{i}}$$

here ${{\varvec{i}}}^{H}$ is Hermitian of ***I*** (current matrix), which can be calculated using Kirchhoff's circuit laws. So ***I*** is supposed to have something to do with the frequency and reactance of each element, depicting as:

8 $${\varvec{i}}=g({f}^{^{\prime}},{\varvec{C}})$$

**Figure 5:**
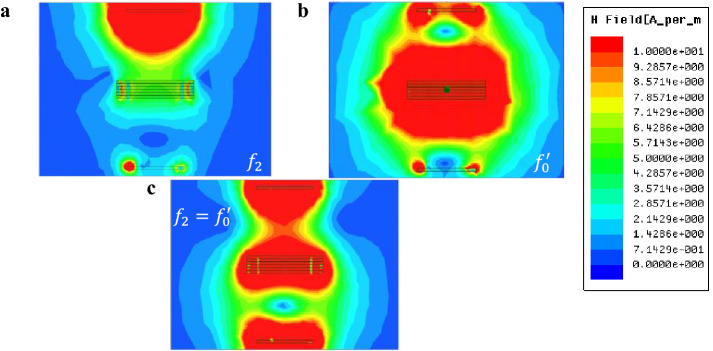
EM simulation on mismatching. (**a**) At the WPT resonant frequency, i.e., the higher band (${f}_{2}$), the wave beam is enforced toward the Rx resulting from the near-zero permeability ($(real\left({\mu }_{z}\right))<1$). At the same time, a low loss is observed when the wave beam go through the metasurface due to the almost-zero characteristics on the imaginary part of permeability ($imag({\mu }_{z})\approx 0$). (**b**) At the metasurface's self-resonance frequency ${f}_{0}^{^{\prime}}$. (**c**) When self-resonance frequency equals the WPT resonance frequency, i.e., higher band, the WPT efficiency significantly increases.

Hence, for each capacitor etched on each element, there must be an optimized ${{\varvec{i}}}^{\boldsymbol{*}}$ in the specific frequency, which means that every time we set a group of value of capacitances, the peak of the WPT efficiency will emerge on the WPT resonance frequency (${f}_{1},{f}_{2}$) and metasurface resonance frequency (${f}_{0}^{^{\prime}}=h({f}_{0})$), respectively, as EM simulation is shown in Fig. 5a,b. Therefore, based on this theory, when we make these two peaks into one by adjusting the capacitors on the Tx, Rx, and metasurface, the two effects are combined, as shown in Fig. 5, a considerable increase in the WPT efficiency. The whole capacitors' adjusting work is relatively more straightforward than the former work^20^. As the capacitance goes higher, the ${f}_{0}^{^{\prime}}$ goes lower simultaneously, resulting from the negative correlation between ${f}_{0}^{^{\prime}}$ and ${f}_{0}$.

### SRRs

The inner and the outer squares can be equivalents of the circle with the identical perimeter shown in Fig. 2a. Therefore, the average radius can be calculated as:

9 $${r}_{0}= \frac{2\times {a}_{0}}{\pi }$$

### The imaginary part of permeability

To estimate the magnetic loss inside the metasurface, we apply the complex Poynting vector, which can be described as:

10 $${\varvec{S}}= \frac{1}{2} Re[{\varvec{E}}\times {{\varvec{H}}}^{\boldsymbol{*}}]$$

**Figure 6:**
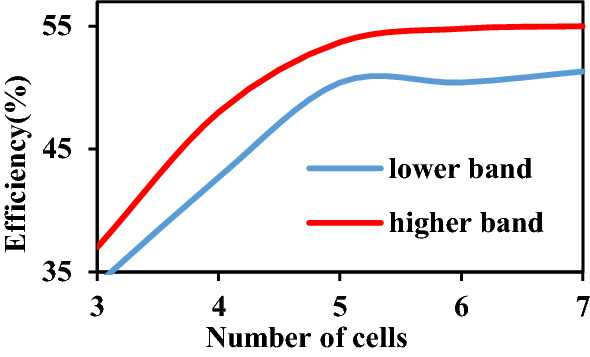
Simulated result of the relationship between the number of cells and efficiency. As the cell of the metasurface increased, the coupling improves until six layers. At the same time, while the layer increases, few efficiencies decreases are estimated, resulting from the almost-zero characteristics on the imaginary part of permeability ($imag({\mu }_{z})\approx 0$).

Therefore, the observed energy ($E$) in the closed region around metasurface (V) can be depicted as^34^:

11 $$ \begin{aligned} W & = - \frac{1}{2}\iint\limits_{s} {{\text{Re}} \left[ {{\varvec{E}} \times {\varvec{H}}^{*} } \right] \cdot d{\varvec{s}}} = - \frac{1}{2}{\text{Re}} \iiint\limits_{V} {div\left( {E \times H^{*} } \right)\;dv} \\ & \quad = \frac{1}{2}\iiint\limits_{V} {\left( {\omega \varepsilon^{\prime\prime}{\varvec{E}} \cdot {\varvec{E}}^{*} + \omega \mu^{\prime\prime}{\varvec{H}} \cdot {\varvec{H}}^{*} + \sigma {\varvec{E}} \cdot {\varvec{E}}^{*} } \right)\;dv} \\ \end{aligned} $$

here, $\varepsilon^{\prime\prime}$ and $\mu^{\prime\prime}$ stands for the imaginary part of permittivity and permeability, respectively. Therefore, when the imaginary part of the permeability is almost zero, the magnetic wave beam can go through the 4.674-mm-thick metasurface over then more with merely low loss, as shown in Figs. 5a and 6.

### EM simulation

The high-frequency simulation software (HFSS) is used for the simulation. The Tx and the Rx are both in the size of 15 mm × 15 mm. The diameter of the inner circle of the Tx (Rx) (${S}_{2}$) is 8 mm, and the diameter of the outer circle ${S}_{2}$ is 12 mm. The size of metasurfaces is 28.8 mm × 28.8 mm and 20 mm × 20 mm for 180° metasurface and 0° metasurface, respectively. Both metasurfaces are used the Rogers 3003 substrate. The width of the coppers (${W}_{copper}$) on metasurfaces are both 1 mm. The space ($space$) between two rings on metasurfaces is both 1.5 mm. The gaps ($space$) for etching capacitors on all the devices are 0.5 mm. The values of the capacitors etched on the 180° metasurface shown in Fig. 2b are all on the inner rings of the SRR with the values (${C}_{lin\mathrm{1,2},\mathrm{3,4},\mathrm{5,6}}$) of 0.3 pF, 0.3 pF, 0.3 pF, 0.3 pF, 0.2 pF, and 0.2 pF. Conversely, the values of the capacitors etched on the 0° metasurface shown in Fig. 2b are all on the outer rings of the SRR with the values (${C}_{hout1,\mathrm{2,3},\mathrm{4,5},6}$) of 0.2 pF, 0.2 pF, 0.2 pF, 0.1 pF 0.1 pF, and 0.1 pF.

**Figure 7:**
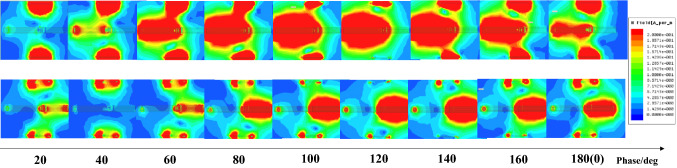
EM simulation of a matched system. The upper and the lower figure stand for the magnetic field at the lower and higher bands, respectively. The WPT efficiency will be maximized simultaneously as the Tx (Rx) and the metasurface resonant separately on each band. Unlike the magnetic field distribution on metasurface resonant itself shown in Fig. 5b, when the self-resonance frequency equals the WPT resonance frequency, the Tx (Rx) will resonant in the different phases.

According to the EM simulation result, only the metasurface resonants, as shown in Fig. 6b, the Tx (Rx), and the metasurface will resonate simultaneously. However, when self-resonance frequency equals the WPT resonance frequency, as shown in Fig. 7, the Tx (Rx) and the metasurface will resonate on different phases.

### Multimode of the proposed metasurface

The proposed metasurface has three modes: the lower mode, the higher mode, and the balanced mode. Since we combined two metasurfaces for the proposed metasurface, when the Tx and Rx are in the area of 180-metasurface, the performance in the higher band maximized and verse versa. Also, when we put the Tx and Rx toward the middle of the metasurface, the enhancement performance on both bands will be equal. Consequently, we categorized those performances as lower-mode, higher mode, and balanced mode, respectively, as shown in Fig. 3d.

### Experiment setup—using a PNA network analyzer

Figures 1d and 3d show the measurement. A network analyzer (PNA series: part #N5222A by Keysight Technologies) is used to measure the proposed WPT system’s S-parameter. The foams and the tapes are used only to fix the whole system, which has few effects on the whole system. Moreover, as a solution to deal with the error that occurred during the capacitors manufacturing and soldering process, the metasurface’s effective frequency is not ideally right on the resonance frequency of the whole system. Air gaps are left and fixed by using foams between each cell of the metasurface. The reason is that when the mutual impedance of each element varies from the size of the gaps, leading to a change on matrix ***Z*** in Eq. (5). In which case, the position of the peak (${f}_{0}^{^{\prime}}$) changes as well. As a result, we can make the ${f}_{0}^{^{\prime}}$ equal to ${f}_{1},{f}_{2}$ easily by changing the size of the air gaps between each cell.

### Experiment setup—using a signal generator and analyzer

**Figure 8:**
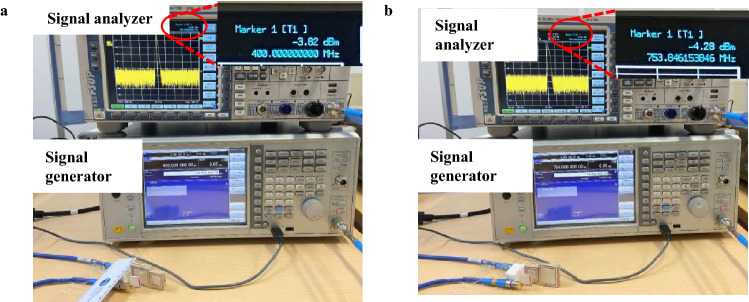
Experiment setup using a signal generator and analyzer, and the results of WPT efficiencies (**a**) in the lower band and (**b**) in the higher band in the WPT distance of 32 mm.

To analyze the proposed WPT system, we also experimented using a signal generator and a signal analyzer, as shown in Fig. 8. The signal generator is connected to the Tx, and the signal analyzer is connected to the Rx. The input power is 0 dBm. We measured the WPT efficiencies of the lower band at lower mode and higher band at higher mode in the WPT distance of 32 mm. The power received in the Rx is − 3.82 dBm at 400 MHz (lower mode) and − 4.28 dBm at 754 MHz (higher mode) in a WPT distance of 32 mm. As shown in Fig. 8, the loss of the cable is − 1.02 dBm at 400 MHz and − 1.12 dBm at 754 MHz. So the actual power that the proposed WPT system transferred is − 2.80 dBm at 400 MHz in the lower mode and − 3.16 dBm at 754 MHz in the higher mode so that the WPT efficiencies are 52% and 48%, respectively. Compared to the WPT efficiency measured by PNA Network Analyzer (54% at 398 MHz and 50.2% at 767 MHz), the WPT efficiency is slightly lower, which maybe because of the peak frequency shift and the loss caused by the SMA connectors. At the same time, the PNA Network Analyzer can eliminate all these losses by performing the calibration before the measurement setup.
