# Author Correction: A multimode metamaterial for a compact and robust dualband wireless power transfer system

**DOI:** 10.1038/s41598-022-09238-1

**Published:** 2022-03-23

**Authors:** Xin Jiang, Ramesh K. Pokharel, Adel Barakat, Kuniaki Yoshitomi

**Affiliations:** Graduate School of Information Science and Electrical Engineering, Kyushu University, Nishi-Ku, Fukuoka, 819-0395 Japan

Correction to: *Scientific Reports* 10.1038/s41598-021-01677-6, published online 11 November 2021

The original version of this Article contained errors in the Acknowledgements section.

“This work was supported in part by JSPS KAKENHI Grant number: 21K04178, in part by the MIC/SCOPE Grant number: 21452069, in part by the Foundation for Technology Promotion for Electronic Circuit Board, and in part by a research granted from The Murata Science Foundation.”

now reads:

“This work was supported in part by the MIC/SCOPE Grant Number: JP215010003, in part by JSPS KAKENHI Grant Numbers: 21K04178, in part by the Foundation for Technology Promotion for Electronic Circuit Board, and in part by a research grant from The Murata Science Foundation.”

The original Article has been corrected.

